# Enhancing faba bean (*Vicia faba* L.) productivity and improving soil fertility using organic amendments in alkaline soil

**DOI:** 10.1186/s12870-026-08541-7

**Published:** 2026-03-13

**Authors:** Hany S. Gharib, Magdy H. Ibrahim, Walid M. El Rodeny, Hend H. Elsharkawy, Diaeldin Omer Abdelkarim, Abouelnadar El Salem, Mahmoud Younis

**Affiliations:** 1 Department of Agronomy, Faculty of Agriculture, University of Kafr Elsheikh, Kafr Elsheikh, 33516 Egypt; 2 Food Legumes Department, Field Crops Research Institute, ARC, Giza, Egypt; 3 Department of Agricultural Engineering, Faculty of Engineering, University of Khartoum, Khartoum, Sudan; 4 Yellow River Delta Intelligent Agricultural Machinery Equipment Industry Academy, Dongying, 257300 China; 5 Chair of Dates Industry and Technology, Department of Agricultural Engineering, College of Food and Agricultural Sciences, King Saud University, PO Box 2460, Riyadh, 11451 Saudi Arabia

**Keywords:** Rice Husk Biochar, Wood vinegar, Biogas Digested Residues, Faba Bean, Alkaline Soil

## Abstract

The present investigation aimed to evaluate the effectiveness of various organic soil amendments in promoting the growth and productivity of faba beans (*Vicia faba* L.) grown in alkaline soil conditions. Eleven treatments were applied on a new faba bean cultivar called Sakha 5 with four separate replicates by using a completely randomized (CRD) experimental design, under a greenhouse pot experiment condition at the Experimental Farm of Sakha Agricultural Research Station, Kafr El-Sheikh, Egypt, during two consecutive seasons from 2021/22 to 2022/23. The amendments included rice husk biochar (RHB), wood vinegar (WV), biogas digested residues (BDR), and molasses (M), applied individually and in six pairwise combinations (WV + RHB, WV + BDR, WV + M, RHB + BDR, RHB + M, and BDR + M). All pots received basal mineral fertilization as calcium superphosphate (to supply 60 g P pot⁻¹), potassium sulfate (to supply 15 g K pot⁻¹), and ammonium nitrate (as an N source, applied in split doses). The data obtained indicated that all soil amendment treatments significantly improved plant growth, seed yield, and protein content in comparison to the unaltered control. Among all treatments, WV + RHB showed the strongest response, raising chlorophyll to 48 and 55 SPAD (control: 42 and 46; ~14–20% increase), root dry weight to 9.67 and 8.30 g plant⁻¹ (control: 5.33 and 3.00; ~56–177% increase), root volume to 10.0 and 13.0 cm³ plant⁻¹ (control: 6.33 and 6.20; ~58–106% increase), and nodulation to 67 and 65 nodules plant⁻¹ (control: 30; ~117–123% increase), while also achieving the highest nutrient uptake (N 3.98–3.68, P 42.10–42.30, K 2.58–2.55 g plant⁻¹; Fe 14.00–14.48, Mn 2.96–2.50, Zn 2.30–2.70 g plant⁻¹) and the maximum seed yield (≈ 37 g pot⁻¹). In conclusion, this research demonstrates that the simultaneous use of rice husk biochar and wood vinegar constitutes a successful and sustainable strategy for improving the productivity of alkaline soils, offering a promising, eco-friendly approach to enhance faba bean crop performance.

## Introduction

In Egypt, faba bean (Vicia faba L.) is a major winter legume whose dry seeds are valued for their high nutritional quality (up to 35% protein and 42–47% carbohydrates) and richness in minerals such as Ca, K, Fe, Mg, and Zn [1]. Due to its symbiosis with rhizobia, faba bean contributes to biological N₂ fixation and can improve soil fertility and function [2]. For example, faba bean may fix up to ~ 200 kg N ha⁻¹, and incorporation of residues can improve soil physical attributes and nutrient status [3]. Egypt has the potential to increase faba bean production because of growing demand from the food sector [4].

Soils with pH > 7 are generally classified as alkaline [5]. High pH and the presence of CaCO₃ typically restrict the availability of essential nutrients (N, P, K, and particularly Fe, Zn, Mn, and Cu) through precipitation, adsorption, and reduced solubility [5, 6]. Alkalinity can also increase NH₃ volatilization losses and decrease phosphorus availability via Ca–phosphate formation [6]. Low organic matter is a common feature of alkaline/calcareous soils, which can further limit productivity [7, 8]. Calcareous and alkaline soils are widespread; for example, calcareous soils are estimated to represent ~ 25–30% of Egypt’s total area, and calcareous soils globally cover nearly one-third of the world’s land surface [9]. Under these conditions, nutrient limitations and reduced microbial activity may constrain crop performance and the efficiency of biological N₂ fixation [5–8]. Faba bean performance can also decline under salt-affected/alkali conditions due to osmotic stress and ion imbalance, with documented yield reductions under increasing salinity [10].

In this context, sustainable soil management increasingly emphasizes organic and recycled amendments because they can improve soil fertility while reducing dependence on intensive mineral fertilizer inputs [11, 12]. Wood vinegar, biochar, and biogas digestate residues can contribute to improving soil chemical and physical quality (e.g., pH buffering, EC moderation, improved aggregation and water retention), while supporting microbial activity and plant tolerance to stress [12, 13]. Molasses, a by-product of sugar processing, is a readily biodegradable carbon source and may stimulate microbial activity and nutrient cycling; it also contains minerals that can support soil fertility, although nutrient availability can remain constrained at high pH [14–19].

Biochar has been widely reported to enhance soil fertility and plant performance, and it also supports climate-change mitigation through carbon stabilization [20–23]. Its high surface area and functional groups can increase cation exchange capacity (CEC), adsorb nutrients, reduce leaching, and provide a favorable habitat for soil microorganisms [24, 25]. Wood vinegar (pyroligneous acid) contains a complex mixture of organic acids and oxygenated organics [26]. Despite this chemical diversity, WV is generally not considered a nutrient-rich input; its direct contribution of mineral nutrients is typically low, so its ability to supply nutrients to plants is limited [27]; however, its organic acids can acidify microsites, chelate metal ions, enhance nutrient solubility, and stimulate beneficial microbial processes, suggesting that combining WV with other amendments may improve performance [28, 29]. Indeed, prior work indicates that combining WV with biochar can provide additional agronomic and environmental benefits [28, 29], and acid-modified biochar has been proposed as a promising strategy for degraded saline-alkaline soils [30].

Biogas digestate residues (BDR) are produced during anaerobic digestion of organic wastes and can be used as biofertilizers or soil conditioners, improving porosity, bulk density, and water retention [31, 32]. Digestates are generally rich in organic matter and plant nutrients and can increase soil N, P, K, and soil organic carbon [33–35]. Because biochar can act as a nutrient-retaining, fertilizer-enhancing matrix in soils, its co-application with digestate or compost may further increase crop yield and productivity [36, 37]. However, comparative evidence on the synergistic performance of WV, rice husk biochar, digestate residues, and molasses—particularly their paired combinations—under alkaline soil conditions remains limited, especially for faba bean–rhizobia systems.

Accordingly, the present study evaluates wood vinegar, rice husk biochar, biogas digestate residues, and molasses—applied singly and in pairwise combinations—on soil nutrient dynamics and the growth, nodulation, yield, and seed quality of the new faba bean cultivar Sakha 5 under alkaline soil conditions. The novelty of this work lies in systematically testing and comparing these locally available organic amendments and identifying the most effective paired strategy for improving alkaline soil fertility and faba bean performance.

## Materials and methods

### Preparation of the soil medium and soil amendments

The soil utilized in the greenhouse study was taken from a rice-growing field at the Sakha Agricultural Research Station Farm. Samples of soil were taken from a field of alkaline-affected soil that was between 0 and 20 cm deep. Wood vinegar (WV) and rice husk biochar (RHB) were obtained from the Central Laboratory for Agriculture Climate (CLAC), Dokki. The RHB was produced by slow pyrolysis using a continuous feeding system under oxygen-limited conditions. The reactor was operated at a set-point temperature of 400 ± 25 °C (monitored by a thermocouple) and the biomass residence time inside the heated zone was 30 min (excluding heating ramp time), which is sufficient for complete carbonization of rice husk under slow-pyrolysis conditions [38, 39]. Biochar formation was confirmed by the production of a stable black carbonized porous residue with constant mass after cooling, indicating completion of devolatilization and conversion to char, while wood vinegar was collected as the condensed liquid fraction of pyrolysis vapors. Molasses (M) was purchased from the Kafr el Sheikh government’s sugarcane factory. The biogas digested residues (BDR) collected from the biogas digester belong to the Ministry of Environment, which is a part of the Kafr El Sheikh government. Fresh cattle manure is used as a raw material in the biogas digester, which is then heated by a solar heating system to maintain a mesophilic temperature of 35 to 40 degrees Celsius. Mechanical systems are used to stir the digesters (at a speed of 20 rpm) every hour. The loading rate is 0–75 m³ per digester per day, with a total volume of 1 m³ per day and an average total solids concentration of 13%, resulting in a retention period of 25 days.

### Soil and soil amendments analysis

Before cultivation, the soil and soil amendments were examined for pH, EC, total organic carbon, and total macro & microelements at the ARC, soil analysis laboratory [40]. Biogas digested residues (BDR), biochar, and soil pH and electrical conductivity (EC) were determined in aqueous suspensions prepared at fixed sample: water ratios (w/v; g dry sample: mL deionized water) of 1:5 (BDR), 1:5 (biochar), and 1:10 (soil), respectively. EC was measured with a conductivity meter (Environmental Express P200, Charleston, SC, USA); Soil pH and amendments under study were measured with a pH meter (Five Easy Plus, Mettler Toledo, Greifensee, Switzerland). Total nitrogen was determined using the Kjeldahl method [41]. According to Jackson [40], total potassium was quantified using a flame photometer, while total phosphorus was assessed calorimetrically using the molybdenum blue (ascorbic acid) method, with absorbance measured by a spectrophotometer.

With a moderate amount of soil organic carbon, the soil had a slightly alkaline pH (Table 1). The total organic C, as shown in Table 2, and total N contents of soil, biochar, and biogas residues were ascertained using a Vario Micro CHNS-O Analyzer (Elementar Analysensysteme GmbH, Hanau, Germany). The utilized molasses was examined for a few chemical characteristics and its essential nutrient content using the techniques outlined by Klute [42] and Page et al. [41]. For chemical analysis of biogas residues, a representative subsample was sent to the Water, Soils, and Environment Research Institute Laboratory, ARC, Giza (Table 1). The primary constituent of wood vinegar was analyzed using GC-MS-QP2010 Shimadzu, Kyoto, Japan, a gas chromatography-mass spectrometer. The fractional injection rate used was 80:1. The electron energy was 70 eV, the ion source temperature was 250 °C, and the mass spectrum scan frequency was 35–400 m z-1. These parameters were maintained for the mass spectrometry. By using retention time and comparing mass spectrometry and mass spectral library data (NIST), compounds were identified. Utilizing the relative peak area, the compound’s content was determined [43].

**Table 1:** Physical and chemical characterization of molasses and Biochar, Biogas, and residues

Attributes and measurements	Soil	Molasses	Biochar	Biogas Digestate Residues	Wood Vinegar
pH	7.9$\pm$0.2	7.6$\pm$0.31	7.8$\pm$0.04	7.2$\pm$0.21	3.5$\pm$0.04
EC^1^ (dS/m)	1.8$\pm$0.03	ND	4.5$\pm$0.01	3.8$\pm$0.04	ND
TOC^2^ (%)	18$\pm0.91$	40$\pm$2.51	48$\pm$3.12	36$\pm$3.54	21$\pm$2.37
OM^3^%	24$\pm$1.1	75$\pm$3.98	ND	66.8$\pm$4.98	ND
Total N %	0.26$\pm$0.005	1.3$\pm$0.01	1.35$\pm$0.05	2.6$\pm$0.04	ND
Ash (%)	ND	12.7$\pm$1.56	ND	45$\pm2.89$	ND
Total Ca	ND	1.2$\pm$0.001	0.26$\pm$0.004	ND	ND
Total Mg (%)	0.28$\pm$0.001	0.8$\pm$0.002	0.21$\pm$0.002	5.7$\pm$0.70	ND
Total Na (%)	0.22$\pm$0.004	0.15$\pm$0.01	0.18$\pm$0.001	ND	ND
Total K (%)	0.03$\pm$0.003	5.3$\pm$0.04	0.23$\pm$0.002	1.2$\pm$0.003	ND
Total P (%)	525$\pm$5.89	1.2$\pm$0.02	0.25$\pm$0.003	0.76$\pm$0.01	ND
Fe (mgKg-1)	68$\pm$3.78	2022$\pm$26	1005$\pm$32.3	1366$\pm$37	ND
Zn(mgKg-1)	74$\pm$8.69	201$\pm$13.2	127$\pm$8.36	192$\pm$7.8	ND
Mn(mgKg-1)	ND	153$\pm$12	110$\pm$9.32	17$\pm$1.08	ND
T. Organic acids (%)	ND	ND	ND	ND	27.1$\pm$1.58
Acetic acid (%)	ND	ND	ND	ND	7.5$\pm$0.67

Values are mean ± SD (n = 3). Abbreviations: *EC* electrical conductivity, *TOC* total organic carbon, *OM* organic matter, *ND* not detected. Units: EC (dS m⁻¹); Fe, Zn, and Mn (mg kg⁻¹)

**Table 2:** Total organic carbon (TOC) under different amendment treatments (g pot⁻¹)

Treatment	M	WV	RHB	BR	M + RHB	M + BDR	M + WV	WV + BDR	RHB + BDR	WV + RHB	
TOC (g pot^− 1^)	10$\pm$0.9	16.8$\pm$1.2	19.2$\pm$1.5	14.4$\pm$0.7	20.9$\pm$2.4	24.4$\pm$3.1	26.8$\pm$1.9	31.2$\pm$0.4	34.8$\pm$2.7	37.4$\pm$3.6	

Values are mean ± SD (*n* = 3). Abbreviations: *TOC* total organic carbon, *WV* wood vinegar, *RHB* rice husk biochar, *BDR* biogas digestate residues, *M* molasses. Treatment rates: WV = 60 mL pot⁻¹; RHB = 20 g pot⁻¹; BDR = 150 g pot⁻¹; M = 50 mL pot⁻¹

### Pot experiment and treatments

A pot experiment was carried out at the Agricultural Research Center, Sakha Research Station, during two winter seasons, 2021/2022 and 2022/2023. The physical characteristics of the soil were clay (35%), silt (28%), fine sand (23%), and coarse sand (13%). It had a clay loam texture. Each 23-liter container was filled with 12 kg of air-dried soil. A completely randomized (CRD) experimental design with factorial arrangements was used to distribute the treatments. There were four duplicates of each treatment. Ten treatment combinations and one control with four replications (a total of forty-four experimental pots) were used. The four soil amendment treatments (1) rice husk biochar (RHB) (2), molasses (M) (3), biogas digested residues (BDR) (4), wood vinegar (WV) were applied at rates of 20 g, 50 ml, 150 g, and 60 ml per pot, respectively. These rates were selected based on a preliminary screening trial and commonly applied ranges in pot experiments to ensure measurable soil improvement without inducing phytotoxicity; accordingly, the applied doses corresponded to RHB = 20 g pot⁻¹ (0.167% w/w; 1.67 g kg⁻¹ soil), BDR = 150 g pot⁻¹ (1.25% w/w; 12.5 g kg⁻¹ soil), molasses = 50 mL pot⁻¹ (4.17 mL kg⁻¹ soil; ≈0.42% v/w assuming density ≈ 1 g mL⁻¹), and WV = 60 mL pot⁻¹ (5.0 mL kg⁻¹ soil; ≈0.50% v/w).

In field-equivalent terms, the biochar dose (20 g pot⁻¹) corresponds approximately to ~ 1.6 t acre⁻¹, which falls within practical application ranges for Egyptian heavy clay soils and was selected to avoid an excessive alkalizing effect of biochar in confined pot conditions [44]. Similarly, the digestate rate (150 g pot⁻¹; ≈12 t acre⁻¹ field-equivalent) represents a typical organic amendment dose used to compensate for low organic matter in clay soils and to provide a sustained nutrient supply. Wood vinegar and molasses were applied at non-toxic bio-stimulant levels, consistent with commonly used aqueous dilutions (≈ 1:200, v/v), to stimulate rhizosphere activity without causing osmotic or acidity stress in potted plants [45].

**Fig. 1:**
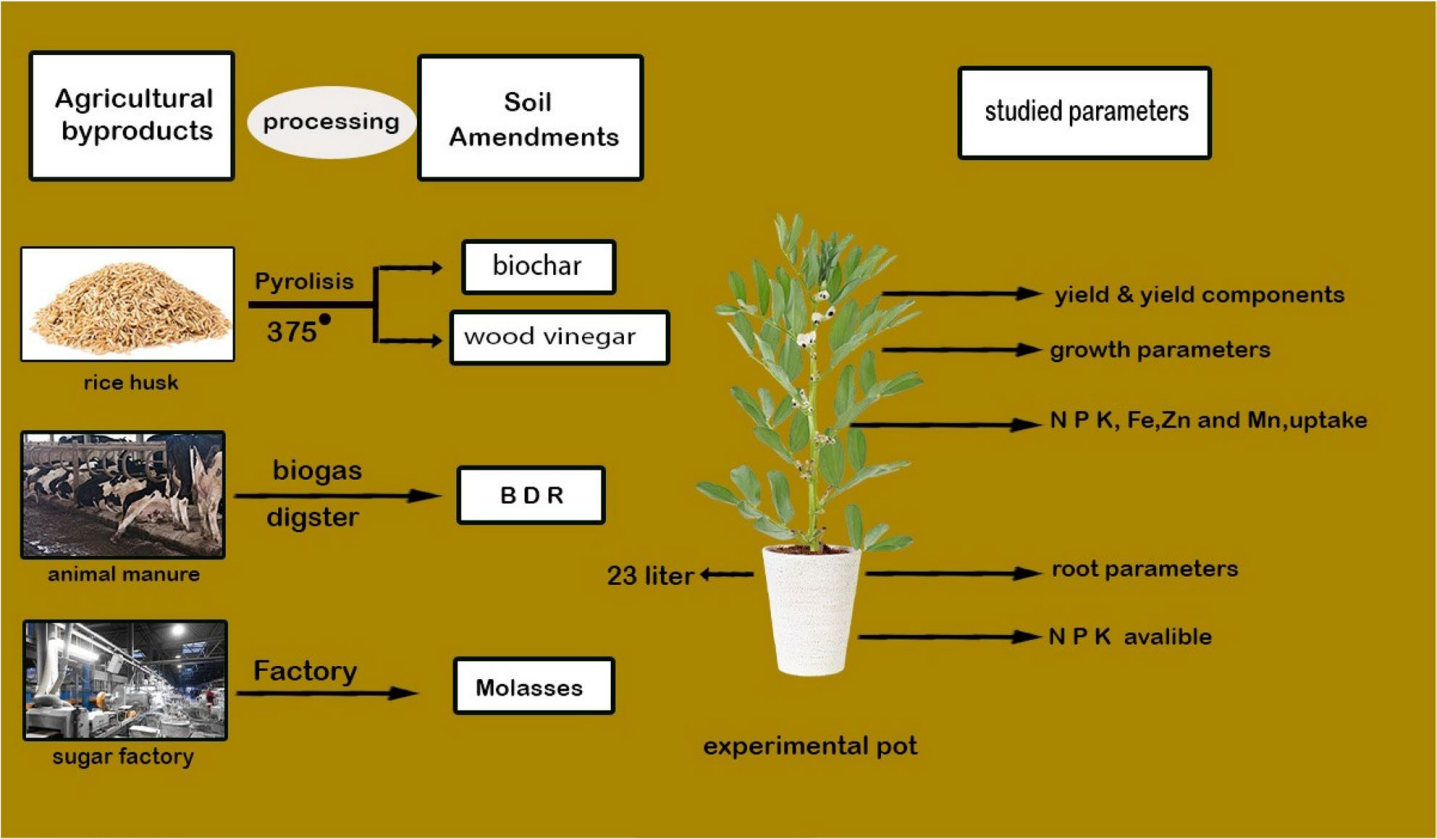
The flowchart of the experiment on the faba bean cultivar Sakha 5

The experimental flowchart is presented in Fig. 1. Superphosphate and potassium sulfate were incorporated into the soil before sowing, and 60 and 15 g of P and K minerals per pot were applied, respectively. Nitrogen was supplied as ammonium nitrate, applied at the recommended rate and split into two equal doses after seedling emergence. Seeds of the broad bean (*V. faba* L.) var. Sakha 5 was obtained from the Legume Research Department, Field Crop Research Institute, ARC, Egypt. A total of 8 seeds of the Sakha 5 cultivar were seeded and subsequently trimmed to three healthy plants per pot following germination. The soil was kept moist at 60% of its water-holding capacity by irrigating the pots with tap water.

### Plant sampling and analysis (chlorophyll content, nodule account, and its fresh weight and root characteristics)

When 50% of the plants were at the flowering stage, one plant of faba bean was harvested from each pot to determine root characteristics, chlorophyll content, and the number of nodules. Using tap water, adhering soil particles were carefully washed from the roots. Each plant’s nodules were taken out, counted, and their dry weight was calculated. Using SPAD (SPAD-502, Sensing Ltd.), the total chlorophyll content of fully expanded leaves was determined. A 100 ml measurement cylinder was used to determine the root volume volumetrically. A plant’s roots were submerged in 50 milliliters of tap water that had been poured into the cylinder, and root volume (cm³) was calculated as the increase in water level after immersion [46]. It was noted that the roots’ immersion caused the water level to rise. The root volume was thought to be represented by the difference between the initial and final volumes. After the samples were oven-dried for 72 h at 70 °C [47], the root dry weight was determined.

### Yield and its constituents

The yield and yield constituents, including the number of branches per plant, the aboveground biomass, and the seed yield per plant, were measured per plant at the end of the physiological maturity stage. Before removing the plant from the pot, the number of branches on each plant was counted inside each pot. Total aboveground dry biomass per plant was determined after harvest and complete sun-drying. After threshing, grain yield per plant was recorded and the seed moisture content was adjusted to 12–15%. Aboveground straw biomass (aboveground biomass excluding grain) was calculated as:

$$\begin{aligned}\begin{aligned}Aboveground\,straw\,biomass\,&=\,total\,aboveground\,dry\,biomass\,\\&-\,grain\,yield\,per\,plant\end{aligned}\end{aligned}$$

### Soil pH and EC, available macro elements, and total organic carbon measurements

Soil samples were collected during harvest from the surface at a depth of 0–30 cm in each pot. Soil pH and electrical conductivity (EC) were determined according to Klute [44]. Available nitrogen (N), phosphorus (P), and potassium (K) were determined following Page et al. [43]. Total organic carbon (TOC) was determined according to Walker et al. [48]. Soil pH, EC, and NPK availability were measured as mentioned before.

### NPK and micronutrient analysis in faba bean plants

After harvesting, faba bean leaves and seeds were dried for 48 h at 70 °C. They were then ground into a fine powder and passed through a 0.5 mm sieve for subsequent analysis. The total concentrations of nitrogen, iron, phosphorus, manganese, potassium, and zinc in the leaves and seeds were determined after a digestion process using a 2:1 ratio of H_2_SO_4_​ and HC_l_O_4_​ [49, 50]. Total crude protein in the seeds was calculated by multiplying the total N percentage by a conversion ratio of 6.25 [51]. The absorption of nitrogen, phosphorus, and potassium by the plants was measured using the method described by Sharma and Behera [52]. Briefly, N, P, and K uptake (absorption) was calculated at harvest by multiplying the nutrient concentration in each plant part (leaves or seeds) by the corresponding dry matter weight (g plant⁻¹), and expressed as mg plant⁻¹ according to: Uptake (mg plant⁻¹) = Concentration (mg g⁻¹ dry matter) × Dry matter (g plant⁻¹). Total plant uptake was calculated as the sum of leaf uptake and seed uptake.

### Experimental design and statistical analysis

The experiment was conducted using a completely randomized (CRD) experimental design with a factorial arrangement, comprising ten treatments and a control, each replicated four times. All data were subjected to statistical analysis of variance (ANOVA) using SPSS v. 21.0 software. Data were presented as mean ± SD based on 3 replicates. Differences among treatment means were assessed utilizing Tukey’s HSD test at a significance threshold of *p* < 0.05 [53]. Linear correlation analysis was performed to evaluate the relationships among total organic carbon, faba bean yield, and soil pH. The data for this analysis consisted of the average values from both years.

## Results

### Effects on soil pH and EC

The application of various amendments significantly influenced soil chemical characteristics, such as pH and EC (Table 3). Across the two experimental cycles (2021/22–2022/23), the combined treatments (WV + RHB, WV + BDR, and M + WV) maintained soil pH in a near-neutral range and were significantly lower than the unamended control. Applying wood vinegar as a single application significantly lowered soil pH compared to the other single treatments, resulting in a decrease of 11% in 2021/22 and by 9.8% in 2022/23 relative to the control. Overall, WV-based combinations consistently showed the strongest pH buffering effect under alkaline conditions.

**Table 3:** The impact of wood vinegar, rice husk biochar, biogas residues, and molasses on N, P, and K availability, and soil pH, EC, and calcium carbonate on the faba bean cultivar Sakha 5

Treatment	Available K (mg Kg^− 1^)		Available *P* (mg Kg^− 1^)		Available Nitrogen (mg Kg^− 1^)		pH		EC		CaCO_3_	
	2021/22	2022/23	2021/22	2022/23	2021/22	2022/23	2021/22	2022/23	2021/22	2022/23	2021/22	2022/23
Control	211.8±7.5c	209.7±9.3d	16.5±0.9e	15.5±1.50d	12.78±0.02d	13.4±0.01d	7.82±0.02d	7.87±1.22c	2.8±0.002e	3±0.001c	9.6$\pm$0.01a	9.8$\pm$0.23a
M	262.3±5.4c	258±8.4c	18.4±1.02c	18.2±0.87c	20.23±0.75c	20±0.6c	7.6±0.01c	7.4±0.84b	2.15±0.001c	2.6±0.002cb	9.3$\pm$0.02a	9.1$\pm$0.9a
WV	310.2±10.6b	259±5.6b	19.9±0.03c	19.6±2b	21±1.2c	20.66±1.6c	7.2±0.6a	7±0.36a	1.7±0.002b	1.8±0.003ab	5.5$\pm$0.01c	5.3$\pm$0.3d
RHB	314±8.2b	267±4.8b	16±0.7d	15.2±0.7d	22.85±1.56b	22.38±2.9b	7.6±0.06c	7.5±0.95b	2.88±0.003e	2.9±0.001c	9$\pm$0.12a	9.2$\pm$0.1a
BDR	317±9.6b	278±12.1b	19.±2.1c	18.3±0.6c	23.86±0.07b	22.9±3.7cb	7.5±1bc	7.4±0.02b	2.4±0.001d	2.2±0.004b	7.5$\pm$0.36b	7$\pm$0.20c
M + RHB	322.4±11.9b	294.1±8.4fb	17.75±0.5c	17±1.1c	25.12±2.5b	25±1.4b	7.55±1.33bc	7.5±0.08b	3.5±0.004f	3.2±0.004d	8.8±0.4a	8.5$\pm$0.82b
M + BDR	333±6.8ab	296±14.5b	21.5±0.86b	22.45±0.9a	25.85±1.78b	25.38±3.7b	7.4±2.65bc	7.40±0.85b	2.50±0.005d	2.88±0.005c	8.2$\pm$0.03b	7.8$\pm$0.23b
M + WV	335±4.3ab	300±3.9b	22.88±1.1a	22.5±0.43a	24.6±0.98b	23.4±1bc	7.3±0.59ab	7.2±0.52a	1.6±0.002b	2±0.001ab	5.7$\pm$0.25c	5.6$\pm$0.7cd
WV + BDR	335.4±8.5ab	320.2±5.4ab	22±2.46a	21.34±1.2a	28.1±1.07a	27.6±1.8ab	7.2±0.06a	7±0.01a	1.4±0.003a	1.5±0.001a	5$\pm$0.01c	5.4$\pm$0.3d
RHB + BDR	353.5±13.1ab	328±2.5ab	21±0.65b	20.34±1.89b	29.23±0.09a	28.86±0.5a	7.44±0.74b	7.44±0.03b	2.4±0.001d	2.3±0.002b	7.2$\pm$0.02b	6.7$\pm$0.70c
WV + RHB	383±10a	342±15.5a	23.3±0.2a	22.6±0.25a	30.6±0.7a	29.6±2.75a	7.2±2.58a	7.1±0.08a	1.5±0.002ab	1.6±0.003a	5.6$\pm$0.10c	5.4$\pm$0.2d
F test	**	**	**	**	**	**	*	**	**	**	**	**
HSD _0.05_	50.12	41.88	1.61	1.72	3.40	3.02	0.23	0.21	0.21	0.55	1.3	1.2

Values are presented as mean ± standard deviation (SD) (*n* = 3). Within each column, means followed by different superscript letters are significantly different at *P* ≤ 0.05 based on Tukey’s HSD test, **=*p* < 0.01. Abbreviations: *WV* wood vinegar, *RHB* rice husk biochar, *BDR* biogas digestate residues, *M* molasses, *EC* electrical conductivity, *CaCO*₃, calcium carbonate, *HSD* honestly significant difference. Treatment rates: WV = 60 mL pot⁻¹; RHB = 20 g pot⁻¹; BDR = 150 g pot⁻¹; M = 50 mL pot⁻¹

Similarly, soil EC showed an overall decreasing trend with WV, both alone and in combination with other amendments. The WV treatment alone reduced soil EC by 39% and 40% in 2021/22 and 2022/23, respectively. The combined application, particularly WV + RHB, produced the most pronounced reduction in EC (up to 46% and 20% across the two seasons), indicating improved control of soluble salts under alkaline soil conditions (Table 3).

Soil calcium carbonate, being one of the key indicators of alkaline soils, showed improvement with the application of WV, both alone and in combination with other amendments. The WV treatment alone reduced soil CaCO_3_ content by 5.5 and 5.3% in 2021/22 and 2022/23, respectively. The combined application, particularly WV + BDR, resulted in a more pronounced reduction, lowering soil CaCO_3_ by 5 and 5.4% compared with the control in the two respective years. On the other hand, there was no significant difference between the WV + BDR and WV + RHB treatments in both seasons. 

### Effects on soil NPK availability

The application of soil amendments, either singularly or in conjunction, markedly influenced the availability of soil macronutrients (Table 3). Overall, treatments containing wood vinegar (WV) consistently produced the greatest improvement in available phosphorus, whether applied alone or in combination, indicating that WV plays a key role in enhancing P solubility under alkaline soil conditions. In contrast, biochar applied alone showed the weakest effect on available P, likely reflecting P precipitation and reduced solubility at higher pH when not buffered by acidic inputs.

For available nitrogen (NH₄⁺–N + NO₃⁻–N), the highest values were generally recorded under combined applications, with WV + RHB ranking among the most effective, followed closely by RHB + BDR and WV + BDR, and no substantial differences among these top treatments were observed (Table 3). Conversely, molasses alone resulted in the lowest available nitrogen, suggesting that C-rich molasses may stimulate short-term microbial immobilization of mineral N when not paired with nutrient-supplying amendments.

A similar pattern was observed for available potassium, where combined treatments—particularly those including RHB—showed the strongest increases. For instance, WV + RHB increased available K from 211.8 (control) to 383.0 mg kg⁻¹ in 2021/22 (+ 171.2 mg kg⁻¹; +80.8%) and from 209.7 (control) to 342.0 mg kg⁻¹ in 2022/23 (+ 132.3 mg kg⁻¹; +63.1%). This agrees with the K-rich ash fraction of rice husk biochar and improved nutrient retention under combined amendment conditions. Overall, the WV + RHB treatment consistently ranked among the top treatments across N, P, and K availability, supporting its superiority in improving nutrient dynamics in alkaline soils.

### Effects on nutrient uptake in faba bean plants

The different amendments significantly influenced the uptake of both macro- and micronutrients by faba bean plants (Table 4). In general, combined treatments outperformed single applications, indicating that improving soil chemical conditions and nutrient availability translated directly into higher plant nutrient acquisition. For macronutrients (N, P, and K), the WV + RHB treatment consistently recorded the highest uptake, while RHB + BDR and WV + BDR ranked second and were statistically comparable to WV + RHB for several traits, confirming that combinations providing both nutrient sources and improved retention/availability are more effective than single amendments. The control consistently exhibited the lowest N, P, and K uptake, reflecting nutrient limitation under alkaline soil conditions.

**Table 4:** The impact of wood vinegar, biogas residues, rice husk biochar, and molasses on N, P, K, Fe, Zn, and Mn uptake of faba bean

Treatment	*N* uptake (g plant^− 1^)		k uptake (g plant^− 1^)		*p* uptake (g plant^− 1^)		Fe uptake (mg plant^− 1^)		Mn uptake (mg plant^− 1^)		Zn uptake (mg plant^− 1^)		
	2021/22	2022/23	2021/22	2022/23	2021/22	2022/23	2021/22	2022/23	2021/22	2022/23	2021/22	2022/23	
Control	1.28$\pm$0.01d	1.32$\pm$0.001 e	1.14$\pm$0.004d	1.18$\pm$0.05d	22.35$\pm$1.22f	22.28$\pm$1.3f	2.98$\pm$0.004c	2.65$\pm$0.05c	0.55$\pm$0.007e	0.73$\pm$0.004c	0.96$\pm$0.005d	1.2$\pm$0.09c	
M	2.28$\pm$0.02c	2.16$\pm$d0.008	1.75$\pm$0.005c	1.68$\pm$0.045c	28.76$\pm$1.05e	29.65$\pm$1.6e	5.6$\pm$0.003b	5.5$\pm$0.02bc	1.2$\pm$0.002d	0.89$\pm$0.002c	1.32$\pm$0.006c	1.56$\pm$0.03bc	
WV	2.45$\pm$0.12c	2.35$\pm$dc0.006	1.78$\pm$0.03c	1.84$\pm$0.33bc	33$\pm$2.80cd	32$\pm$d0.90c	6.5$\pm$0.008b	6.7$\pm$0.08b	1.33$\pm$0.007d	1.03$\pm$0.003c	1.65$\pm$0.002a	1.83$\pm$0.007bc	
RHB	2.62$\pm$0.006c	2.58$\pm$c0.003	1.97$\pm$0.06bc	2$\pm$0.021b	31$\pm$2.64d	30$\pm$0.84dc	3.56$\pm$0.004c	3.76$\pm$0.02c	0.76$\pm$0.001e	0.78$\pm$0.003c	1.06$\pm$0.003c	1.32$\pm$0.02cb	
BDR	2.64$\pm$0.03c	2.63$\pm$c0.004	2.18$\pm$0.07b	2.11$\pm$0.014b	35$\pm$1.85c	35.4$\pm$0.23bc	6.2$\pm$b0.02	5.6$\pm$0.02bc	1.23$\pm$0.001d	1$\pm$0.001c	1.4$\pm$0.001c	1.7$\pm$0.04bc	
M + RHB	3.2$\pm$0.18b	2.7$\pm$c0.008	2.38$\pm$0.55a	2.3$\pm$0.052a	32$\pm$3.80d	33$\pm$1.80c	5.62$\pm$b0.03	5.42$\pm$0.08bc	1.15$\pm$0.001d	0.88$\pm$0.002c	1.17$\pm$0.008b	1.5$\pm$0.07bc	
M + BDR	3.22$\pm$0.05b	2.95$\pm$bc0.002	2.43$\pm$0.003a	2.37$\pm$0.007a	36.60$\pm$0.85bc	36.48$\pm$2.5b	7.30$\pm$b0.09	8.50$\pm$0.05bc	1.5$\pm$0.003c	1.54$\pm$0.001b	1.86$\pm$0.007ab	2$\pm$0.001b	
M + WV	3.29$\pm$0.02b	3.15$\pm$0.001 b	2.47$\pm$0.041a	2.42$\pm$0.008a	36.74$\pm$0.6b	36.65$\pm$4.2b	11.44$\pm$0.08ab	10.96$\pm$0.03ab	2.2$\pm$0.002b	1.8$\pm$0.002b	1.9$\pm$0.005ab	2.3$\pm$0.05ab	
WV + BDR	3.32$\pm$0.01a	3.25$\pm$0.07 ab	2.49$\pm$0.020a	2.52$\pm$0.008a	39$\pm$4.70b	38.4$\pm$3.80b	13.5$\pm$1a	14$\pm$0.02a	2.21$\pm$0.004ab	2$\pm$0.003ab	2.2$\pm$a0.02	2.66$\pm$0.12a	
RHB + BDR	3.45$\pm$0.04a	3.66$\pm$0.032 a	2.54$\pm$0.36a	2.53$\pm$0.002a	42$\pm$4.20a	41.27$\pm$0.54a	8.95$\pm$0.52bc	8.68$\pm$b0.08c	1.67$\pm$0.07c	1.70$\pm$0.004b	1.75$\pm$0.007bc	1.97$\pm$0.004bc	
WV + RHB	3.98$\pm$a0.21	3.68$\pm$0.04 a	2.58$\pm$0.078a	2.55$\pm$0.047a	42.1$\pm$1.05a	42.3$\pm$2.98a	14$\pm$0.98a	14.48$\pm$0.12a	2.96$\pm$0.08a	2.50$\pm$0.005a	2.30$\pm$0.003a	2.70$\pm$0.002a	
F test	**	**	**	**	*	**	**	**	**	**	**	**	
HSD_0.05_	0.67	0.44	0.31	0.34	2.47	2.78	4.72	4.88	0.77	0.55	0.65	0.55	

Values are presented as mean ± standard deviation (SD) (*n* = 3). Within each column, means followed by different superscript letters are significantly different at *P* ≤ 0.05 based on Tukey’s HSD test, **=*p* < 0.01. Abbreviations: *WV* wood vinegar, *RHB* rice husk biochar, *BDR* biogas digestate residues, *M* molasses, *N* nitrogen, *P* phosphorus, *K* potassium, *Fe* iron, *Zn* zinc, *Mn* manganese, *HSD* honestly significant difference. Treatment rates: WV = 60 mL pot⁻¹; RHB = 20 g pot⁻¹; BDR = 150 g pot⁻¹; M = 50 mL pot⁻¹

Regarding micronutrients (Fe, Mn, and Zn), WV + RHB also produced the strongest overall improvement, followed by WV + BDR and M + WV, showing that treatments incorporating WV were particularly effective in enhancing micronutrient uptake.

### Effects on plant growth and yield traits

All amendments, particularly the combined applications, significantly improved faba bean growth and yield traits compared to the control (Tables 5 and 6). Overall, combined treatments consistently outperformed single applications, indicating that integrating amendment functions (acidification/chelation by WV, nutrient supply by BDR, and retention/CEC improvement by RHB) enhances rhizosphere performance and plant development. Among all treatments, WV + RHB produced the strongest and most stable improvements across both seasons, reflected by higher chlorophyll (SPAD), stronger root development (root dry weight and root volume), and markedly increased nodulation relative to the control (Table 5). Compared with the control, WV + RHB increased chlorophyll (SPAD) by 14.3% (2021/22) and 19.6% (2022/23); root dry weight by 81.4% and 176.7%; root volume by 58.0% and 109.7%; nodule fresh weight by 108.3% and 110.5%; and nodule number by 123.3% and 116.7% in 2021/22 and 2022/23, respectively. These improvements are consistent with better nutrient acquisition and enhanced Rhizobium activity under moderated alkaline conditions (Tables 3, 4 and 5).

**Table 5:** Effect of wood vinegar, rice husk biochar, biogas residues, and molasses on chlorophyll content, root traits, and nodulation of faba bean

Treatment	Chlorophyll		Dry weight of roots/plant		Root volume/plant		Fresh weight of nodules/plant		Number of nodules/plants	
	2021/22	2022/23	2021/22	2022/23	2021/22	2022/23	2021/22	2022/23	2021/22	2022
control	42$\pm$2.59b	46$\pm$2.54c	5.33$\pm$0.01c	3$\pm$0.03c	6.33$\pm$0.03c	6.2$\pm$0.06e	4$\pm$0.003c	3.80$\pm$0.002c	30$\pm$1.35e	30$\pm$2.64e
M	42$\pm$3.61b	50$\pm$6.50b	6.33$\pm$0.06c	5.5$\pm$0.02b	6.67$\pm$0.05c	7.47$\pm$0.02d	4.3$\pm$0.004c	4.3$\pm$0.003b	40$\pm$1.06d	39±3.55d
WV	43$\pm$1.2b	52$\pm$2.58a	7.33$\pm$0.04bc	6$\pm$0.08b	8$\pm$0.11b	8$\pm$0.04d	4.3$\pm$0.001c	4.83$\pm$0.002b	44$\pm$2.30c	40$\pm$0.52cd
RHB	44$\pm$0.95b	52$\pm$0.65a	7.5$\pm$0.03bc	6.03$\pm$0.09b	8$\pm$0.13b	8.13$\pm$0.14d	4.33$\pm$0.003c	5.2$\pm$0.001b	45$\pm$1.66c	41.33$\pm$1.56cd
BDR	44$\pm$4.96b	53$\pm$1.54a	8.0$\pm$0.05b	6.50$\pm$0.06b	8.33$\pm$0.04b	9.67$\pm$0.13c	5$\pm$0.021c	5.5$\pm$0.002b	51.33±3.46b	44$\pm$2.87cd
M + RHB	45$\pm$0.65a	53$\pm$2.69a	8.33$\pm$0.06b	6.77$\pm$0.05b	8.33$\pm$0.06b	9.83$\pm$0.06c	5.3$\pm$0.02cbd	5.67$\pm$0.005b	53.67$\pm$2.98b	45$\pm$2.33c
M + BDR	45$\pm$5.97a	54$\pm$1.74a	8.33$\pm$0.02b	7$\pm$0.07a	9$\pm$0.32a	10.33$\pm$0.05b	6.3$\pm$0.03b	5.73$\pm$0.012b	54.67$\pm$1.89b	47.33$\pm$4.55b
M + WV	46$\pm$3.25a	54$\pm$3.56a	8.67$\pm$0.03a	7$\pm$0.01a	9$\pm$0.26a	10.57$\pm$0.03b	7.3$\pm$0.045a	6.3$\pm$0.001a	55.$\pm$4.65b	47.67$\pm$3.88b
WV + BDR	46$\pm$4.78a	54$\pm$2.86a	9$\pm$0.07a	7.33$\pm$0.02a	9$\pm$0.14a	11$\pm$1.03b	7.33$\pm$0.031a	6.3$\pm$0.002a	55$\pm$3.98b	52$\pm$2.88b
RHB + BDR	47$\pm$5.60a	55$\pm$4.56a	9.3$\pm$0.03a	7.6$\pm$0.03a	9.67$\pm$0.07a	11.67$\pm$2.03b	7.67$\pm$0.023a	6.7$\pm$0.001a	55$\pm$2.54b	54.33$\pm$2.36b
WV + RHB	48$\pm$1.73a	55$\pm$0.25a	9.67$\pm$0.06a	8.3$\pm$0.05a	10$\pm$0.09a	13$\pm$2.04a	8.33$\pm$0.031a	8$\pm$0.003a	67$\pm$3.66a	65$\pm$4.58a
F test	*	*	**	**	**	**	**	**	**	**
HSD_0.05_	3.96	3.66	1.21	1.31	1.68	1.04	1.15	1.37	4.73	5.23

Values are presented as mean ± standard deviation (SD) (*n* = 3). Within each column, means followed by different superscript letters are significantly different at *P* ≤ 0.05 based on Tukey’s HSD test, **=*p* < 0.01. Abbreviations: *WV* wood vinegar, *RHB* rice husk biochar, *BDR* biogas digestate residues, *M* molasses, *SPAD* soil-plant analysis development (chlorophyll meter reading), *HSD* honestly significant difference. Treatment rates: WV = 60 mL pot⁻¹; RHB = 20 g pot⁻¹; BDR = 150 g pot⁻¹; M = 50 mL pot⁻¹

**Table 6:** Effect of wood vinegar, rice husk biochar, biogas residues, and molasses on yield and yield components of faba bean

Treatment	Yield plant^− 1^		Weight of the biological yield plant^− 1^		Number of pods plant^− 1^		Protein (%)		Number of branches plant^− 1^	
	2021/22	2022/23	2021/22	2022/23	2021/22	2022/23	2021/22	2022/23	2021/22	2022/23
Control	17.33$\pm$1.25e	24.33$\pm$2.56g	49.67$\pm$2.33e	57.67$\pm$0.66f	6.7$\pm$0.03d	7.3$\pm$0.07c	20$\pm$0.98d	18.51$\pm$1.68d	2$\pm$0.001b	1.73$\pm$0.001c
M	19$\pm$1.33d	36.33$\pm$1.62f	53.33$\pm$1.62d	75$\pm$0.36e	8.3$\pm$0.01c	7$\pm$0.06c	21.46$\pm$0.65d	18.76$\pm$0.96dc	2$\pm$0.002b	2.$\pm$0.001bc
WV	24.3$\pm$2.36c	37$\pm$3.56ef	60$\pm$2.56c	79.33$\pm$2.23dc	9$\pm$0.07c	7$\pm$0.03c	23.54$\pm$1.56c	19$\pm$0.26dc	2.2$\pm$0.001b	2.2$\pm$0.001bc
RHB	25$\pm$3.47b	38$\pm$3.59e	61.33$\pm$0.66c	80$\pm$0.25dc	9$\pm$0.08b	7.5$\pm$0.07bc	23.96$\pm$2.58c	19.78$\pm$0.65c	2.33$\pm$0.003b	2.21$\pm$0.002bc
BDR	25.33$\pm$3.65b	39$\pm$0.96de	62.33$\pm$1.33bc	80$\pm$7.65dc	10.7$\pm$0.06b	7.8$\pm$0.09b	24.33$\pm$1.54b	20.94$\pm$0.79bc	2.23$\pm$0.001b	2.2$\pm$0.003bc
M + RHB	26$\pm$3.56b	40$\pm$4.5cd	63.33$\pm$0.67bc	80.67$\pm$5.63dc	10.7$\pm$0.03b	7.8$\pm$0.13b	24.38$\pm$1.33b	21.58$\pm$0.95b	2.33$\pm$0.002a	2.4$\pm$0.003a
M + BDR	26.33$\pm$2.46b	41$\pm$0.36c	64$\pm$2.45ab	81$\pm$1.54cb	11$\pm$1.02ab	8$\pm$0.23ab	25$\pm$2.57b	21.63$\pm$1.79b	2.67$\pm$0.001a	2.43$\pm$0.001a
M + WV	26.33$\pm$0.96b	43$\pm$2.85b	64.67$\pm$0.67ab	84.33$\pm$2.35bc	11$\pm$1.02ab	8$\pm$0.26ab	25.83$\pm$1.36b	21.83$\pm$1.26b	2.67$\pm$0.002a	2.6$\pm$0.001a
WV + BDR	27.8$\pm$0.89a	45$\pm$3.89a	64.67$\pm$4.63ab	89$\pm$2.87a	11.7$\pm$1.05ab	8.2$\pm$0.53ab	30.58$\pm$3.59a	23.49$\pm$2.61a	2.67$\pm$0.001a	2.63$\pm$0.002a
RHB + BDR	28$\pm$2.89a	45$\pm$3.54a	65$\pm$1.33ab	90$\pm$6.53a	12$\pm$0.30a	8.3$\pm$0.38a	30.83$\pm$1.07a	24.02$\pm$2.58a	2.67$\pm$0.002a	2.7$\pm$0.002a
WV + RHB	29$\pm$0.96a	45$\pm$1.63a	66$\pm$1.67a	93$\pm$4.23a	12.3$\pm$0.33a	8.3$\pm$0.23a	32.08$\pm$2.59a	24.94$\pm$0.98a	2.67$\pm$0.003a	2.73$\pm$0.001a
F test	**	**	**	**	**	**	**	**	*	*
HSD_0.05_	1.73	1.54	2.06	3.61	1.35	0.47	1.5	1.7	0.44	0.50

Values are presented as mean ± standard deviation (SD) (*n* = 3). Within each column, means followed by different superscript letters are significantly different at *P* ≤ 0.05 based on Tukey’s HSD test, **=*p* < 0.01. Abbreviations: *WV* wood vinegar, *RHB* rice husk biochar, *BDR* biogas digestate residues, *M* molasses, *HSD* honestly significant difference. Treatment rates: WV = 60 mL pot⁻¹; RHB = 20 g pot⁻¹; BDR = 150 g pot⁻¹; M = 50 mL pot⁻¹

Yield Traits (Table 6): All combined applications under study consistently enhanced yield parameters. The WV + RHB treatment produced the most significant improvements, increasing plant yield (16.1–17.8%) and (13.7–13.3%), biological yield weight (9.1–14.7%) and (7.1–14.0%), number of pods (15.97–27.00%) and (21.1–9.6%), protein content (23.82–26.62%) (25.3–20.7%), and number of branches (19.50–25.00%) (12.7–19.0%) compared to the single treatment of WV alone and RHB alone in both years respectively. Although some single treatments (WV, RHB, and BDR) also improved yield compared to the control, their effects were not significantly different from each other. The concurrent administration of (WV) and (RHB) proved to be the most effective. This mixture consistently improved soil properties, boosted nutrient uptake, and led to substantial rises in the growth and output of faba bean plants.

### Relationship between soil pH, faba bean yield, and soil organic carbon

**Fig. 2:**
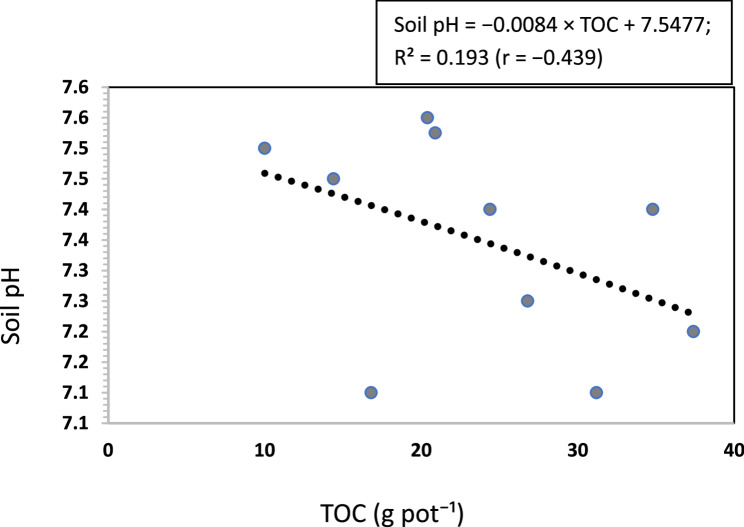
The relationship between TOC and soil pH. Abbreviations: TOC, total organic carbon; r, Pearson correlation coefficient; R², coefficient of determination

**Fig. 3:**
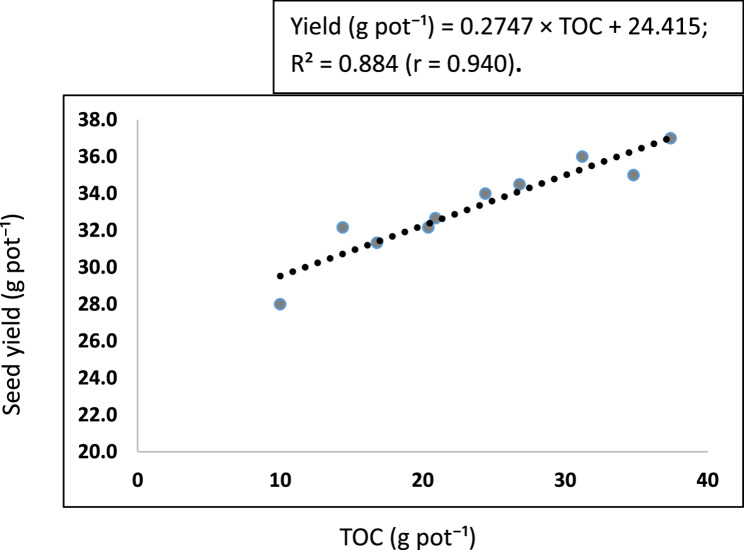
The relationship between TOC and soil yield. Abbreviations: TOC, total organic carbon; r, Pearson correlation coefficient; R², coefficient of determination

The correlation analysis (Fig. 2) showed that the relationship between TOC and soil pH indicated a moderate positive linear relationship (r = $-$0.439; R² = 0.193). This suggests that increasing TOC tended to slightly decrease soil pH, although TOC explained only 19.3% of the variation in soil pH. In the WV + RHB treatment, TOC reached 37.4 g pot⁻¹, which was associated with a lower soil pH. The faba bean yield was significantly positively correlated with SOC (*r* = 0.940; R² = 0.884). The highest yield of 37 g pot⁻¹ was recorded when the pot was amended with WV + RHB (Fig. 3).

## Discussion

This study evaluated the comparative effects of wood vinegar (WV), rice husk biochar (RHB), biogas digestate residues (BDR), and molasses (M), both individually and in combination, on soil chemical properties, nutrient dynamics, and the growth and productivity of faba bean. (*Vicia faba* L.).

### Effect of single and combined soil amendment treatments on soil EC and pH

The integrated application of RHB, WV, BDR, and molasses was hypothesized to exert varying influences on soil quality and faba bean performance. The intrinsic properties of these materials, specifically the high carbon content of biochar, the nutrient-dense profile of biogas residues, and the organic acid-rich nature of wood vinegar, are expected to trigger synergistic or antagonistic effects [25, 29]. Such outcomes arise from complex interactions governing nutrient bioavailability and microbial proliferation within the soil matrix.

Regarding electrical conductivity (EC), the influence of soil amendments was contingent upon the specific material combinations. As a carbon-rich amendment, RHB introduces soluble alkaline salts and ash, which typically elevate soil EC; the release of basic cations (e.g., K^+^, Ca^+ 2^, and Mg^+ 2^) further increases the concentration of soluble ions in the soil solution [53]. In contrast, WV, an acidic byproduct of biomass pyrolysis, is abundant in organic acids, phenols, and trace minerals.

When WV is applied in combination with RHB, the organic acids can act as chelating agents, complexing with metal ions and mitigating the potential EC increase from biochar alone, especially in saline or alkaline soils [54]. This aligns with the findings of Wu et al. [55], who discovered that a combined WV and biochar treatment significantly lowered soil EC and dissolved organic carbon. This reduction in EC may be attributed to complementary mechanisms reported in [27]: wood vinegar can acidify the soil solution and stimulate cation exchange, which promotes displacement of exchangeable Na⁺ by Ca²⁺/Mg²⁺, while biochar provides a high-surface-area, high-CEC matrix that can adsorb and retain soluble ions. In parallel, the combined application can improve soil aggregation and permeability, enhancing infiltration and facilitating leaching of soluble salts, thereby lowering the salt concentration in the soil solution [29]. In addition to direct chemical interactions, this combined application enhances soil properties that indirectly affect EC, such as boosting microbial activity and modifying the concentrations of other soluble nutrients [27].

Ultimately, the outcome of biochar amendment on soil EC is complex, depending on initial soil conditions, biochar properties, feedstock type, and application rates [55]. The dynamic between the alkaline nature of biochar and the acidic compounds of wood vinegar leads to a nuanced regulation of soluble salts and nutrients, which generally remains within a range conducive to crop development [56].

The findings of this research demonstrated that the use of RHB alone slightly elevated soil pH and water-soluble salt content compared with other treatments. The notable rise in soil pH observed with RHB alone was primarily attributed to the hydration of exchangeable sodium (forming NaOH) and the hydrolysis of CO₃²⁻ and HCO₃⁻ ions, which consume H⁺ and produce OH⁻ [57].

Conversely, the wood vinegar used in this investigation had an intrinsic pH of 3.5, containing 7.5% acetic acid. Wang and El-Sawy [58] observed that soil pH under WV application alone decreased significantly and continued to decline with increasing doses. Unlike strong mineral acids, WV organic acids degrade readily within the soil. Consequently, the synergistic use of WV and RHB substantially mitigated the increase in soil pH induced by RHB alone, reducing the salinity associated with biochar applied independently [59].

Furthermore, in alkaline soils, when calcium carbonate acts as a binder, organic acids in wood vinegar react with CaCO_3_ to break it down, which reflects its effects on soil properties [30, 60].

### Effect of single and combined soil amendment treatments on the availability of NPK in soil and their uptake in faba bean plants

Our current study shows that the combined use of wood vinegar (WV) and rice husk biochar (RHB) markedly enhanced the availability and uptake of nitrogen (N), phosphorus (P), and potassium (K) in faba bean plants [61]. This combination resulted in higher NPK availability and uptake compared to single applications, with no significant differences observed between WV + RHB, WV + BDR, and RHB + BDR treatments, except for phosphorus, which was positively affected by all treatments incorporating wood vinegar [61]. This trend likely reflects the acidifying and chelating actions of WV that improve nutrient solubility under alkaline conditions, while biochar and/or digestate enhance nutrient retention and rhizosphere functionality, thereby facilitating uptake.

The observed increases in N, P, and K concentrations in faba bean plants may be attributable to the sustained release of nutrients from these combined treatments, providing necessary elements throughout the growing season [62, 63]. Biochar and biogas residuals (BDR) act as reservoirs that retain and gradually release nutrients, while simultaneously stimulating microorganisms and plant roots to secrete phytohormones and metabolites within the rhizosphere [62, 63].

Furthermore, the enhanced solubilization of nitrogen and various micronutrients and their uptake in faba bean plants under the combined treatments (WV + RHB, WV + BDR, and RHB + BDR) may also be linked to improvements in biological N₂ fixation and/or the biosynthesis of organic acids. Decreased nitrification rates and altered soil enzyme activity levels [61, 64]. Consequently, the synergistic effects of the WV + RHB treatment not only increased faba bean seed yield but also amended and sustained alkaline soils through the improvement of soil health, including pH stabilization and the increased availability of macro- and micronutrients [61].

The combined use of RHB, BDR, and WV also markedly enhanced soil organic carbon and acid content. When WV or BDR is combined with RHB, the high organic carbon level of the biochar promotes soil aggregate formation, producing a synergistic effect [65], although the mineralization of these components may reduce over time [66]. While all materials—separately or collectively—improved NPK availability, the synergistic use of RHB and WV proved to be more efficacious than other combinations or singular applications.

Specifically, the organic acids in WV promote nitrogen release and transform stable soil phosphorus into more labile forms. This mechanism stimulates microbial growth and expedites the dissolution of phosphorus from biochar [37], therefore elevating the levels of hydrolysable nitrogen and available phosphorus in the soil [67]. While the RHB and WV treatment was the most effective, the combined BDR and WV application ranked second, which is consistent with BDR directly supplying nitrogen and phosphorus.

### Effect of single and combined soil amendment treatments on the *Rhizobium* parameters, growth traits, and yield and its components of faba bean

The application of these amendments and their combinations significantly and positively influenced faba bean growth traits (chlorophyll content, root dry weight, and root volume plant⁻¹), Rhizobium parameters (nodule fresh weight and number plant⁻¹), and yield components (yield plant⁻¹, biological yield weight, number of pods, and branches plant⁻¹, along with protein percentage). These overall trends agree with the growth and yield patterns summarized in Tables 5 and 6, where combined treatments clearly outperformed the control and most single applications.

The WV + RHB combination was the most successful treatment in increasing faba bean quality, yield, and growth. This finding implies that, especially in alkaline soil, the combined WV + RHB treatment improves soil chemical conditions and nutrient accessibility. The enhanced faba bean growth and yield under the combined WV + RHB treatment is probably the result of the two substances interacting favorably. Together, these factors increase the availability and uptake of essential nutrients (NPK), stabilize soil pH, and promote healthier root and nodule growth. Consequently, photosynthetic capacity is likely enhanced, as indicated by the higher chlorophyll (SPAD) values recorded under the WV + RHB treatment.

The beneficial effects are also linked to the high concentration of organic acids (phenols, furans, ketones, and aldehydes) in WV, which boost the activity of beneficial soil microbes and, in addition to acting as a biostimulant, encourage the plant to produce its own hormones (such as IAA) [68]. It also contains chemical precursors such as tryptophan, which is converted inside the plant into a growth hormone [69, 70]. Organic acids in WV stimulate the metabolic activity of Rhizobium bacteria, thereby increasing nitrogen fixation [71]. This interaction explains the increased chlorophyll content, as prior studies have demonstrated a robust link between plant nitrogen content and chlorophyll levels [72]. Furthermore, the significant improvement in N and P availability directly contributes to higher chlorophyll content, promoting overall development.

Biochar, as a carbon-rich substance, is essential for improving soil structure, water retention, soil microorganisms, and nutrient holding capacity [73]. By improving soil porosity, surface area, and cation exchange capacity (CEC) [56, 74]. Research indicates that the simultaneous application of WV with RHB promotes nutrient uptake and improves overall soil quality [75]. Consistent with these mechanisms, combined WV + RHB treatment was superior to single-application or control groups.

The acetic acid in WV contributes significantly to this synergistic effect by encouraging microbial expansion [56, 76]. Soil microbes and plant roots convert acetic acid into Acetyl-coenzyme A (acetyl-CoA), a building block for substances that support growth. Additionally, biochar aids in climate change mitigation by facilitating carbon storage and diminishing greenhouse gas emissions, such as N_2_ and CH_4_ [56, 68]. The synergistic effect of WV and RHB stimulates both physical and biosynthetic activities, improving plant physiological functions and the uptake of macro- and micronutrients.

Similar benefits have been observed in other systems; for instance, combining WV and manure biochar can improve tomato plant growth and yield through improved soil aggregation, nutrient recycling, and phytohormone synthesis [74, 77, 78].

Finally, our study demonstrated that plants treated with combinations (WV + RHB, WV + BDR, and RHB + BDR) had higher protein content. Taken together, these results confirm that combined organic amendments, particularly WV + RHB, reinforce the functional link between Rhizobium activity, vegetative growth, and final yield in faba bean grown under alkaline soil conditions [79, 80].

### Effect of single and combined soil amendment treatments on the Fe, Zn, and Mn uptake in faba bean plants

Applying rice husk biochar (RHB), wood vinegar (WV), biogas residues (BDR), and molasses either separately or in combination affects the uptake of micronutrients by faba beans in different ways. The combined effect of the individual application of WV and RHB enhanced soil structure and microbial activities, while the application of WV rapidly increases the availability of Fe, Zn, and Mn through soil acidification and chelation via its functional groups, although this effect may be more persistent than that of chemical acids. Rice husk biochar application has long- term benefits over WV and chemical amendments, but its effect on micronutrient availability is heavily reliant on the initial pH of the soil [78, 81].

Despite the benefits of single applications, the combined use of RHB + WV and BDR + WV is likely superior, where it works in concert to address the problems arising from the application of treatments individually, as mentioned above. This synergistic interaction is essential for creating effective soil management plans that improve faba bean nutrition and advance sustainable farming.

### Correlation effect of SOC on soil pH and faba bean yield

Organic carbon acts as a primary buffer for soil pH. Organic matter provides a buffering effect for soil pH and increases the soil’s ability to tolerate sharp changes in soil pH. In alkaline soils, organic matter acts as a chelating complex for nutrients, which helps increase the availability of micronutrients. In alkaline soils (such as most of Egypt’s soils), the release of organic carbon helps to form organic acids and carbon dioxide, which contribute to controlling soil pH within the appropriate range [77, 80]. There is a negative correlation between increased soil pH and both soil organic carbon and final yield. Consequently, the relationship between organic matter and the final yield is positive. Appropriate soil pH improves the status of phosphorus and micronutrients, making them more readily available for absorption. On the other hand, by increasing the SOC, soil aeration and water-retention capacity are improved; this mitigates water stress and prevents harmful effects on the plant, especially during its sensitive stages [81].

## Conclusions

The findings indicate that the utilization of organic amendments, including rice husk biochar (RHB), wood vinegar (WV), biogas digestate residues (BDR), and molasses (M), significantly improved the yield, growth traits, nutrient assimilation, and protein concentration of the faba bean cultivar Sakha 5 under alkaline soil conditions. Among the tested treatments, WV + RHB showed the strongest overall performance, followed by WV + BDR and then BDR as the most effective single amendment. The superior response under WV + RHB suggests a synergistic effect that can enhance soil fertility and promote NPK availability/uptake, which may support Rhizobium activity and plant development. Overall, the study supports WV + RHB (and, to a slightly lesser extent, WV + BDR) as a practical strategy to improve faba bean productivity in alkaline soils by reducing soil pH and electrical conductivity. Enhanced soil pH and faba bean yield were positively associated with increased soil organic carbon content; however, since this work was conducted under greenhouse pot conditions using one soil source and one cultivar, field validation across different alkaline soils and environments is recommended, alongside optimization of WV: RHB rates/ratios and multi-season testing to confirm the consistency of these benefits.

## Data Availability

The datasets generated and/or analyzed during the current study are available from the corresponding author on reasonable request.
